# Poster Session I - A161 G0S2 PROTEIN REGULATES COLON INFLAMMATION AND COLORECTAL CANCER THROUGH PRODUCTION OF BRANCHED-CHAIN FATTY ACIDS

**DOI:** 10.1093/jcag/gwaf042.161

**Published:** 2026-02-13

**Authors:** Y Wang, R Zagani, O Chen, M De Sa Tavares Russo, D Avizonis, A Bessissow, J Teodoro

**Affiliations:** Biochemistry, McGill University, Montreal, QC, Canada; Harvard Medical School, Boston, MA; Biochemistry, McGill University, Montreal, QC, Canada; Biochemistry, McGill University, Montreal, QC, Canada; Biochemistry, McGill University, Montreal, QC, Canada; Biochemistry, McGill University, Montreal, QC, Canada; Biochemistry, McGill University, Montreal, QC, Canada

## Abstract

**Background:**

Branched-chain fatty acid esters of hydroxy fatty acids (FAHFAs) are a novel class of endogenous anti-inflammatory and anti-colitis lipids. It has recently been discovered that adipose triglyceride lipase (ATGL), a key enzyme in triglyceride lipolysis, catalyzes the synthesis of FAHFAs. The G0S2 protein (G0/G1 switch gene 2) functions as an endogenous inhibitor of triglyceride breakdown by binding to ATGL. However, how the regulation of FAHFA production by the ATGL/G0S2 axis affects colon inflammation and colorectal cancer progression has never been addressed.

**Aims:**

This study aims to investigate how the ATGL/G0S2 axis regulates the production of anti-inflammatory FAHFAs and how this regulation impacts colon inflammation and colorectal cancer.

**Methods:**

We have previously derived a *G0s2* knockout (KO) mouse and we measured FAHFA levels *in vivo* by Liquid Chromatography-Mass Spectrometry (LC-MS). To assess the impact of FAHFAs on inflammation, we treated the *G0s2* KO and wild-type controls with DSS to induce experimental colitis. Additionally, we used AOM/DSS-induced colorectal tumorigenesis and *Apc*^*Min/+*^-driven intestinal cancer model to examine how G0S2 affects tumour progression.

**Results:**

We found that *G0s2* KO mice are more resistant to DSS induced colitis and both AOM/DSS induced and *Apc*^*Min/+*^ driven tumorigenesi.

**Conclusions:**

Our data suggest that the ATGL/G0S2 axis can affect colon inflammation and colorectal cancer through ATGL-mediated FAHFA production.

**Funding Agencies:**

FRQS, FRQNT, CODE LiFE Research Award, CRS

